# The Effect of Chitosan Edible Coating Containing Nanoemulsion of *Eryngium campestre* Essential Oil on the Quality of Ostrich Meat During Storage in Refrigerator

**DOI:** 10.1002/fsn3.70237

**Published:** 2025-05-10

**Authors:** Faegheh RoshanNejad Moadab, Maryam Azizkhani

**Affiliations:** ^1^ Department of Food Hygiene, Faculty of Veterinary Medicine Amol University of Special Modern Technologies Amol Iran

**Keywords:** chitosan, edible coating, *Eryngium campestre*, meat preservation, nanoemulsion, ostrich

## Abstract

The objective of this study was to determine the effect of chitosan edible coating containing *Eryngium campestre* (zulang) essential oil (EO) nanoemulsion on the quality of ostrich meat during refrigerated storage. Main components of the EO included germacrene D, campestrolide, salvial‐4(14)‐en‐1‐one, and α‐bisabolol. The zulang nanoemulsion droplets had an average diameter of 75 nm and a zeta potential of −32 mV. The samples were coated with chitosan and chitosan incorporated with 1%, 2.5%, and 5% of zulang EO nanoemulsion, stored at 4°C for 12 days, and physicochemical (free fatty acid (FFA), peroxide value (PV), pH, total volatile basis nitrogen (TVB‐N), drip loss, and texture), and microbial analysis were carried out. Samples treated with chitosan/2.5% or 5% EO nanoemulsion had significantly lower FFA, PV, TVB‐N content, and microbial (mesophiles, Enterobacteriaceae, psychrotrophs) counts during cold storage in comparison to the samples coated with pure chitosan and control. Also, the treatments containing chitosan and 2.5% or 5% EO nanoemulsion showed lower shear force, higher texture tenderness, and lower drip loss. The treatments' efficiency as antimicrobial agents and maintaining physicochemical properties were as follows: chitosan/5% EO nanoemulsion > chitosan/2.5% EO nanoemulsion > chitosan/1% EO nanoemulsion > chitosan. The findings suggest that combining chitosan with EO nanoemulsion enhances its interaction with the food matrix, facilitating better dispersion and sustained release of the EO, which ensures a more uniform antimicrobial effect throughout the food product.

## Graphical abstract

Ostrich meat samples coated with chitosan containing nanoemulsion of *Eryngium campestre* essential oil had lower free fatty acid, peroxide value, and total volatile bases and also lower microbial count. Chitosan coatings incorporated with 2.5% and 5% nanoemulsion of *Eryngium campestre* essential oil decreased the drip loss and increased the texture tenderness of the samples.

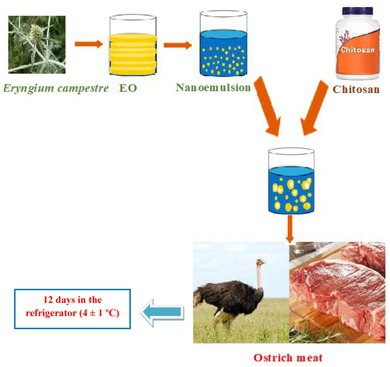

## 1 Introduction

Meat and meat products are considered to be highly perishable foods, and their contamination by pathogenic microorganisms can cause food poisoning. The growth of bacteria, along with biochemical and enzymatic changes, causes spoilage in this type of food products (Lázaro et al. 2015). In recent years, the increase in the production of ostrich meat and its numerous nutritional benefits has led consumers to welcome this type of meat (Rad et al. 2018). Ostrich ( *Struthio camelus* ) is the largest living bird belonging to the ratite family and is raised in most countries of the world for meat production. Ostrich meat has many advantages over other livestock meats, so it has been introduced as the superior red meat and the meat of the 21st century. The sensory characteristics of ostrich meat, such as texture and taste, are very similar to beef and are popular with consumers (Poławska et al. 2011). Ostrich meat is a lean, nutrient‐rich red meat with significantly lower fat, collagen, and saturated fatty acid content compared to chicken and turkey. Its low fat and collagen levels are particularly important, as they contribute to slower spoilage rates and make it a suitable candidate for natural preservation strategies (Heydari et al. 2020). Tenderness is one of the prominent features of ostrich meat, which is due to the low amount of saturated fatty acids and the low ratio of collagen to protein. The low amount of collagen makes it easy to digest and chew better. Ostrich meat does not lose much water during cooking, which ensures a crispy and juicy texture of the meat. The use of the edible part of the carcass is limited to the thigh, and there is not much difference in the content of fatty acids, cholesterol, and fat in ostrich muscles (Poławska et al. 2011). The shelf life of ostrich meat at refrigerator temperature and in conventional packaging is 3 days (Heydari et al. 2016), but in general, various types of modern packaging are used to maintain quality and increase the shelf life of food (Khodaman et al. 2022). Currently, there is a growing interest in the use of edible coatings and films and natural antioxidants in research works and also in industry due to their potential to improve quality and shelf life of food products (Shokraneh et al. 2017). Today, essential oils (EOs) and plant extracts have been considered as natural preservatives or food additives with antioxidant and antimicrobial properties. As uncontrolled use of additives carries the possibility of causing toxicity and their adverse interaction with food components, the use of active edible films and edible coatings with controlled release rates of active ingredients can be useful in addressing concerns and increasing product shelf life without negatively affecting their sensory properties (Ranjbaryan et al. 2019; Mosallaie et al. 2024). Edible coatings can be applied to foods in various ways, the two most common of which are: (1) edible coatings that are applied directly to the food product, and (2) pre‐formed films that are wrapped around the food product (Suhag et al. 2020). EOs are natural compounds rich in terpenes, extracted from various plant parts through distillation or steam extraction (Ramsey et al. 2020). *Eryngium campestre (zulang*), native to northern Iran, is a medicinal plant with recognized therapeutic and edible applications. Its EO contains bioactive compounds such as monoterpenes, flavonoids, polyphenols, and saponins, which contribute to its antimicrobial and antioxidant activities (Nebija et al. 2009; Tit and Bungau 2023). Recent studies have demonstrated that *E. campestre* EO exhibits broad‐spectrum antimicrobial activity, including efficacy against foodborne pathogens such as *Escherichia coli, Staphylococcus aureus*, and *Listeria monocytogenes* (Thiem et al. 2010; Kikowska et al. 2016). Its notable antioxidant properties are attributed to its high content of flavonoids and polyphenolic compounds. While EOs such as basil ( *Ocimum basilicum* ), thyme ( *Thymus vulgaris* ) and oregano ( *Origanum vulgare* ) are widely recognized for their antimicrobial and antioxidant activities and have been extensively studied and applied in food preservation (Boskovic et al. 2015; Sakkas and Papadopoulou 2017), *E. campestre* remains relatively underexplored in this context. Its promising bioactivity, unique phytochemical profile, and regional availability make it a novel and valuable candidate for developing natural preservation strategies in food systems.

EO coatings, while recognized for their antimicrobial and antioxidant properties, often face challenges such as poor water solubility, volatility, strong aroma, and potential degradation under environmental stresses (light, heat, and oxygen). These limitations can reduce their efficacy and limit their application in food systems. Recent advances, particularly the use of nanoemulsion‐based EO coatings, have addressed many of these challenges. Nanoemulsions, due to their small droplet size and increased surface area, enhance the dispersion and stability of EOs in aqueous environments. They improve the controlled release of bioactive compounds, reduce the strong sensory impact of EOs, and increase their interaction with microbial membranes, leading to improved antimicrobial activity. For instance, Mehraie et al. (2023) demonstrated that nanoemulsion‐based EO coatings provided more uniform coverage and sustained antimicrobial activity on food surfaces, compared to conventional EO coatings (Mehraie et al. 2023). Thus, nanoemulsion technology serves as a promising strategy to overcome the limitations of traditional EO coatings, enhancing their functional performance in food preservation applications.

Chitosan is a deacetylated product of chitin that is widely used in the pharmaceutical and food industries due to its biodegradable, biocompatible, and less toxic nature (Singh et al. 2019).

Chitosan nanoparticles not only enhance the physical stability and bioavailability of active ingredients by forming a controlled release system but also exhibit emulsifying and crosslinking capabilities. The nanoscale particle size increases the surface area‐to‐volume ratio, improving water solubility, colloidal stability, and the sustained release of encapsulated compounds. When combined with *E. campestre* EO, these properties help protect its volatile components, prolong its antimicrobial and antioxidant effects, and improve its dispersion in food systems. Studies have shown that nanoemulsion‐based delivery systems can significantly enhance EO efficacy (de Pinho Neves et al. 2014; Wang and Zhuang 2022). Reports on the application of biopolymers for encapsulating bioactive compounds using biopolymers for packaging using immersion, spraying, and other film production methods on fresh meat slices, meat and fish products (Alemán et al. 2016; Alizadeh Behbahani and Imani Fooladi 2018; Yang et al. 2023), fish fillets (Choulitoudi et al. 2016; Valipour Kootenaie et al. 2017), beef, lamb, and chicken (Riquelme et al. 2017; Chang et al. 2019; Behbahani et al. 2020, 2024; Garavito et al. 2020) have been published; but to the best of our knowledge, no previous research has investigated the preservation of ostrich meat using a biopolymer‐based coating incorporating *E. campestre* EO in nanoemulsion form. Therefore, the aim of this study was to determine the effect of chitosan edible coating containing *E. campestre* EO nanoemulsion on the quality of ostrich meat during refrigerated storage. The novelty lies in the use of the nanoemulsified EO, which is expected to enhance the bioavailability, controlled release, and stability of the bioactive compounds, offering superior preservation effects compared to non‐nanoemulsified systems.

## 2 Materials and Methods

### 2.1 Chemicals and Culture Media

All chemicals and culture media used in this study were purchased from Merck (Germany), and their respective CAS numbers are as below: Sodium chloride (CAS No. 7647‐14‐5), peptone (CAS No. 73049‐73‐7), agar (CAS No. 9002‐18‐0), tripolyphosphate (sodium tripolyphosphate, CAS No. 7758‐29‐4), 2‐Thiobarbituric acid (TBA) (CAS No. 504‐17‐6), Trichloroacetic acid (TCA) (CAS No. 76‐03‐9), sodium hydroxide (NaOH, CAS No. 1310‐73‐2), hydrochloric acid (HCl, CAS No. 7647‐01‐0), Tween 80 (polysorbate 80, CAS No. 9005‐65‐6), ethanol (CAS No. 64‐17‐5), chitosan (CAS No. 9012‐76‐4), sodium thiosulphate (CAS No. 7772‐98‐7), phloroglucinol (CAS No. 108‐73‐6), Nutrient Agar (containing peptone and beef extract, CAS numbers for components: peptone, CAS No. 91079‐98‐6; beef extract, CAS No. 68909‐34‐6), MRS Agar (De Man, Rogosa, and Sharpe Agar, containing peptone, beef extract, glucose, sodium acetate, magnesium sulfate, and manganese sulfate, CAS numbers: peptone, CAS No. 91079‐98‐6; beef extract, CAS No. 68909‐34‐6; glucose, CAS No. 50‐99‐7; sodium acetate, CAS No. 127‐09‐3; magnesium sulfate, CAS No. 7487‐88‐9; manganese sulfate, CAS No. 7785‐87‐7), Violet Red Bile Agar (VRBG, containing crystal violet, CAS No. 548‐62‐9; bile salts (Sodium taurocholate, CAS No. 139‐64‐8), glucose, CAS No. 50‐99‐7, and agar, CAS No. 9002‐18‐0) and Plate Count Agar (PCA, containing peptone and sodium chloride, CAS numbers: peptone, CAS No. 91079‐98‐6; sodium chloride, CAS No. 7647‐14‐5).

### 2.2 Plant Preparation and Essential Oil Extraction

Fresh *E. campestre* plant was purchased from the local market of Sari city (Mazandaran province) and transferred to the Food Chemistry Laboratory of Amol University of Special Modern Technologies. Having been cleaned and washed, the leaves were dried in an oven (Model HOT‐789, GHI Technologies, France) at 40°C; then the EO was extracted using a Clevenger apparatus (Model CLEV‐456, DEF Scientific Equipment, USA) for 4 h. The EO was stored in a dark glass until use.

### 2.3 Identification of Essential Oil Compounds

A gas chromatograph (Thermo Quest 2000, UK) equipped with a mass spectrometer (GC/MS) was used to identify the EO compounds. The GC was fitted with an HP‐5MS column (30 m × 0.25 mm × 0.25 μm) and the carrier gas was helium (He) with a flow rate of 1.2 mL/min. The temperature gradient started at 50°C, held for 2 min, then ramped at 5°C/min to 250°C, and was held for 5 min. N‐alkanes (C8–C20) were used as reference points for calculating the relative retention indices (RRI), with the data also compared to reference books and standard libraries (Wiley 275.L and Wiley 7n.L) (Adams 2007). Quantification was performed using the peak area % method, with no internal standard used (Adams 2007).

### 2.4 Ostrich Meat Preparation

Fresh ostrich thigh meat was purchased from a local farm in Semnan city (Semnan province) and transferred to the Food Chemistry Laboratory of Amol University of Special Modern Technologies in a box containing ice.

### 2.5 Preparation of Chitosan Solution Containing Essential Oil Nanoemulsion

The chitosan solution was prepared using the ionic gelation method. In this method, commercial chitosan powder with an average molecular weight and a viscosity of approximately 200–800 mPa·s was dissolved in 100 mL of 1% (w/v) acetic acid solution. Then, 4 mL of 2% tripolyphosphate solution was added to 100 mL of chitosan solution that was being stirred on a magnetic stirrer (Model XYZ‐123, ABC Instruments, Germany) (stirring speed 200 rpm). The stirring continued for 60 min at 50°C ± 1°C to completely dissolve the chitosan. The pH of the chitosan solution was adjusted to 4–5 using NaOH or HCl (Liang et al. 2024). To prepare EO nanoemulsion, the EO was mixed with Tween 80 (as surfactant) in the ratio of 1:1 (v/v) and ethanol (as co‐surfactant and 10% v/v of Tween 80). The emulsification was carried out by gradually adding water under vigorous stirring, followed by ultrasonic treatment using an ultrasonic device (UP400S model, Hielscher Co., Germany) with a power of 1.5 kW, amplitude of 80%, and a pulse duration of 0.5 s for 10 min. To ensure the formation of nanoemulsion, particle size, zeta potential, and dispersion index were determined using a Nanozetasizer (model ZEN 3600, Malvern, UK) at a wavelength of 363 nm. To incorporate the EO nanoemulsion into the chitosan solution, the prepared nanoemulsion was added to the chitosan solution under continuous stirring at 200 rpm for 10 min (nanoemulsion concentrations: 1%, 2.5%, and 5% v/v), until a homogeneous mixture was achieved, ensuring uniform dispersion of the nanoemulsion within the chitosan matrix (Ehyaeirad et al. 2024).

### 2.6 Sample Treatment With Chitosan Containing EO Nanoemulsion

The ostrich meat was cut into 200 g pieces with approximate dimensions of 10 × 5 × 5 cm and treated in the following groups: Control group: no edible coating; F1: Group treated with chitosan solution (2% chitosan and 2% tripolyphosphate); F2: Group treated with chitosan solution containing 1% of EO nanoemulsion; F3: Group treated with chitosan solution containing 2.5% of EO nanoemulsion; F4: Group treated with chitosan solution containing 5% of EO nanoemulsion. Ostrich meat pieces were immersed in the prepared solution groups for 15 min and then placed on a sterile metal dripper for 10 min. The pieces were vacuum‐sealed individually in sterile zip‐packs, and all samples were stored at a temperature of 4°C ± 1°C for 12 days. The samples were subjected to physicochemical and microbial evaluations at intervals of every 3 days.

### 2.7 Chemical Tests

#### 2.7.1 Chemical Analysis of the Ostrich Meat

Chemical analysis of the ostrich meat was carried out before treatment to determine the fat content, protein, mono‐unsaturated fatty acids, poly‐unsaturated fatty acids, and vitamin B_12_, according to AOAC methods (AOAC, 2016).

#### 2.7.2 pH Value

Ten grams of the fillet samples were added to 25 mL of neutral distilled water, homogenized (model Ultra Turrax T25 Digital Homogenizer, IKA‐Werke GmbH & Co. KG in Germany) kept for 10 min at room temperature, and filtered using Whatman paper no. 1. The pH was determined using a pH meter (model HI98103, Hanna Instruments, USA) (Rahman et al. 2015).

#### 2.7.3 Free Fatty Acid Content (FFA)

FFA value was determined according to the method of Rahman et al. (2015). Five grams of the fillet sample was homogenized in 30 mL of chloroform at 11000 *g* for 1 min and then filtered using Whatman filter paper no. 1 to remove fillet particles from the filtrate. After adding four to five drops of ethanolic phenolphthalein (1%) as an indicator, the filtrate was titrated with ethanolic potassium hydroxide solution, and the FFA content on the basis of oleic acid was calculated as follows:

$$\mathrm{FFA}\;\left(\%\right)=\left(V\times N\times 28.2\right)/W$$

where “*V*” is the volume of titration (mL) with KOH, “*N*” is the normality of the KOH solution, and “*W*” is the sample weight (g) (Rahman et al. 2015).

#### 2.7.4 Total Volatile Base Nitrogen (TVB‐N)

TVB‐N compounds are the sum of primary, secondary, and tertiary amines in the form of volatile amines and toxic nitrogen compounds, which are used as the biomarker of protein and amine degradation and spoilage. These toxic compounds have considerable adverse effects on the organoleptic properties and acceptability of meat products and increase during the storage period of the product. To determine TVB‐N, 10 g of fillet sample, 2 g magnesium oxide (MgO), and 500 mL of distilled water were transferred into a balloon, and volatile nitrogen compounds were accumulated in a solution of boric acid (2%) and methyl red (as the indicator). Titration of the solution was carried out with sulfuric acid, and the results were reported as mg TVB‐N/100 g of chicken fillet according to the following equation (Jonaidi Jafari et al. 2018):

TVN=sulfuric acid×14

#### 2.7.5 Peroxide Value (PV)

Peroxide value is an indicator of the concentration of hydroperoxides, which are primary oxidation products formed during the initial stages of lipid oxidation. An increase in PV indicates early oxidative changes in meat. The PV was determined according to the method explained by Rahman et al. (2015). Ten grams of the samples were weighed in a 250‐mL Erlenmeyer flask and heated at 60°C for 3 min in a water bath to melt the fat. Then, 30 mL acetic acid‐chloroform solution (3:2 v/v) was added and the flask was thoroughly agitated for 2 min to dissolve the fat. To separate tissue particles from the liquid part, the suspension was filtered using Whatman filter paper no. 1; 0.5 mL of saturated potassium iodide solution and four to five drops of starch solution (as the indicator) were added to the filtrate. The solution was titrated against the standard solution of sodium thiosulfate. PV was expressed as milliequivalent peroxide per kilogram of the sample and calculated by the following equation:

$$\mathrm{PV}\;\left(\mathrm{meq}/\mathrm{kg}\right)=\left(V\times N\right)/W\times 100$$

where “*V*” is the volume of titration (ml), “*N*” is the normality of sodium thiosulfate solution, and “*W*” is the sample weight (g) (Rahman et al. 2015).

#### 2.7.6 Thiobarbituric Acid Reactive Substances (TBARS)

TBA reactive substances indicate secondary oxidation products, mainly malondialdehyde (MDA), which are responsible for rancid odors and flavors. TBARs provide a reliable indicator of advanced lipid degradation. The TBARs assay was conducted following the protocol outlined by De Leon and Borges (2020). Briefly, meat samples were homogenized with TCA to precipitate proteins and release malondialdehyde (MDA). After centrifugation, the supernatant was reacted with TBA under acidic conditions and heated to form a pink chromogen. The absorbance of this complex was measured at 532 nm using a spectrophotometer. The TBARs values were expressed as milligrams of MDA per kilogram of meat (De Leon and Borges 2020).

### 2.8 Microbial Tests

To prepare decimal dilutions of fillet samples, 10 g of each sample was homogenized with 90 mL of 0.1% peptone water using a stomacher (BagMixer 400 SW, HealthCare Technologies Co., Cape Town, South Africa).

#### 2.8.1 Total Mesophilic Bacteria

For enumerating total mesophilics, the decimal dilutions prepared above were inoculated onto the PCA medium and the plates were incubated (model INCU‐Line, Abtron Equipment Ltd., UK) at 37°C for 24 h. The result of plate counting was reported as log_10_ cfu/g and was performed in triplicate (Mahdavi et al. 2018; Kukhtyn et al. 2020).

#### 2.8.2 Psychrotrophic Bacteria

To evaluate the growth of psychrotrophic bacteria, samples from decimal dilutions above were cultured on PCA and incubated at 7°C for 10 days. The results were reported as log_10_ cfu/g and performed in triplicate (Mahdavi et al. 2018; Kukhtyn et al. 2020).

#### 2.8.3 Enterobacteriaceae

VRBG agar was used to culture and count Enterobacteriaceae (in triplicate) upon incubation at 37°C for 24 h. The counting results were expressed as log_10_ cfu/g (Mahdavi et al. 2018; Kukhtyn et al. 2020).

#### 2.8.4 Lactic Acid Bacteria

To investigate the growth of lactic acid bacteria (LAB), MRS agar was used. The incubation condition was 30°C for 2 days. All counts were expressed as log_10_ cfu/g and performed in triplicate (Mahdavi et al. 2018; Kukhtyn et al. 2020).

### 2.9 Drip Loss

Drip loss is water escaping from raw poultry meat during storage. Drip loss was measured by suspending 50‐gram chicken fillet samples individually in polyethylene bags without any touch with the sides of the bags for 24 h at 4°C. Then, samples were removed from the bags, gently dried, and weighed. Drip loss was determined as the percentage of weight lost (Rahman et al. 2015):

$$\begin{array}{c} \text{Drip loss}\;\left(\%\right):\\ \left[\left(\text{sample weight}\;\left(g\right)-\text{sample weight after}\;24\;h\;\left(g\right)\right)/\text{sample weight}\;\left(g\right)\right]\\ \times 100 \end{array}$$

### 2.10 Texture Analysis

The texture of the samples was analyzed for firmness by peak shear force (*g*) applying a texture analyzer instrument (TA.XTplusC, Stable Microsystems Co., Surrey, UK). Texture softness/firmness was determined by the shear energy (*N* × mm). Ten fillet samples (*n* = 10) of each treatment were tested, and the readings were carried out in triplicate (Khan et al. 2022).

### 2.11 Sensory Evaluation

Sensory evaluation was performed to determine the odor, flavor, color, tenderness, and overall acceptability of ostrich meat samples during refrigerated storage. A 9‐point hedonic scale was employed, where 1 = dislike extremely and 9 = like extremely, following standardized sensory analysis protocols. A panel of 10 trained individuals (aged 22–45 years), previously selected based on their experience and interest in meat product evaluation, participated in the sensory tests. Panelists received training to familiarize themselves with the hedonic scale and specific sensory attributes of ostrich meat using reference samples to ensure scoring consistency and accuracy. Meat samples (approximately 2 × 2 × 2 cm) were cooked using an electric grill to an internal temperature of 75°C ± 1°C and allowed to cool slightly before serving. Each sample was coded with a random three‐digit number and presented to panelists in a randomized order under controlled lighting and environmental conditions. Panelists were provided with water for palate cleansing between samples (Yarali 2023).

### 2.12 Statistical Analysis

All tests were performed in triplicate, and the data obtained in the study were presented as mean ± standard deviation. Data analysis was performed to compare the antimicrobial and antioxidant activities of the edible coating containing the nanoemulsion and the edible coating without the nanoemulsion during the storage period using the two‐way analysis of variance (ANOVA) statistical test. All statistical tests were performed at a confidence level of 95%. Tukey's HSD post hoc test was applied for multiple pairwise comparisons to identify significant differences between treatment groups.

## 3 Results and Discussion

### 3.1 Chemical Composition of Ostrich Meat

The chemical composition of ostrich thigh meat, as presented in Table 1, highlights its high nutritional value, characterized by a low fat content of 1.02% (w/w) and a high protein level of 21.85% (w/w). Additionally, it contains substantial amounts of mono‐unsaturated fatty acids (39.03% w/w fat), poly‐unsaturated fatty acids (27.61% w/w fat), and vitamin B12 (12.9 μg/kg). Compared to conventional meats, ostrich meat offers notable health benefits. Beef typically contains higher fat levels (10%–20% depending on the cut), and while chicken is leaner, it still has higher fat than ostrich in most parts, especially with the skin on. Moreover, ostrich meat provides similar or higher levels of heme iron and vitamin B12 compared to beef, while maintaining a healthier fat profile (Zdanowska‐Sąsiadek et al. 2018). This makes ostrich meat a favorable alternative for consumers seeking nutrient‐dense, low‐fat red meat.

**TABLE 1:** The chemical composition of ostrich thigh meat.

Composition	Amount	Composition	Amount
Dry matter (w/w%)	23.67	Fat (w/w%)	1.02
Protein (w/w%)	21.85	SFA^a^ (w/w fat%)	33.30
Ash (w/w%)	1.15	MUFA^b^ (w/w fat%)	39.03
B12 (μg/kg)	12.9	PUFA^c^ (w/w fat%)	27.61

^a^ Saturated fatty acid.

^b^ Mono‐unsaturated fatty acid.

^c^ Poly‐unsaturated fatty acid.

### 3.2 Essential Oil Components

GC–MS analysis resulted in the identification of 39 components for *E. campestre* EO (Table 2). Main components of the EO included germacrene D (36.90%), campestrolide (18.54%), salvial‐4(14)‐en‐1‐one (4.55%), and α‐bisabolol (4.25%). As presented, the main compound of *E. campestre* EO in this work is composed of terpenes and terpenoids, and the other of aromatic and aliphatic constituents. Campestrolide has been found by Medbouhi et al. (2018) as a new uncommon and naturally found 17‐membered ring lactone in *E. campestre* EO that possesses conjugated acetylenic bonds and cytotoxic activity (Medbouhi et al. 2018). Our results confirm the earlier report of Medbouhi et al. (2019) that major volatile constituents obtained from the aerial parts of *Eryngium campestre* were germacrene D (53.4%), Campestrolide (35.3%), and Germacrene B (21.5%) (Medbouhi et al. 2019). The lower content of germacrene D in our study may be attributed to factors such as plant origin, environmental conditions, harvesting time, or extraction method, all of which can influence EO composition. These differences may affect the EO's bioactivity profile, especially its cytotoxic and antimicrobial potential. Also, Arabpoor et al. (2021) reported that the main components of *Eryngium campestre* were germacrene, which has been reported to possess strong antimicrobial activity (Medbouhi et al. 2019; Arabpoor et al. 2021).

**TABLE 2:** Chemical composition of *Eryngium campestre* essential oil identified by GC/MS.

Compounds	Amount (%)	Retention Index	Compounds	Amount (%)	Retention Index
β‐Pinene	Tr	973	Sesquicineole	0.55	1507
Myrcene	1.61	982	β‐curcumene	0.43	1510
p‐Cymene	Tr	1011	δ‐cadinene	Tr	1513
Limonene	Tr	1020	δ‐cadinene	0.4	1515
(Z)‐β‐Ocimene	Tr	1026	(*E*)‐α‐bisabolene	1.07	1533
δ‐Terpinene	Tr	1045	β‐Elemol	Tr	1534
Nonan‐2‐one	Tr	1077	7‐epi‐trans‐Sesquisabinene hydrate	0.35	1546
Terpinolene	Tr	1080	Salvial‐4(14)‐ene‐1,5‐epoxide	0.84	1549
Nonanal	Tr	1084	Germacrene B	0.19	1551
Decanal	0.25	1173	Spathulenol	0.66	1536
α‐Copaene	0.49	1379	Caryophyllene oxide	0.81	1571
β‐Elemene	2.83	1391	Salvial‐4(14)‐en‐1‐one	4.55	1578
β‐Ylangene	0.75	1420	Ledol	0.46	1604
δ‐Elemene	Tr	1429	1,10‐di‐epi‐Cubenol	1.38	1611
(E)‐β‐Farnesene	2.3	1450	α‐Cadinol	1.57	1646
α‐Curcumene	0.51	1473	α‐Bisabolol	4.25	1666
Germacrene D	36.9	1480	14‐Hydroxy‐α‐muurolene	0.6	1760
β‐Selinene	0.13	1485	14‐Hydroxy‐δ‐cadinene	0.71	1785
α‐Muurolene	2.05	1504	Campestrolide	18.54	2144
β‐Bisabolene	Tr	1504	Total	84.28	

### 3.3 Nanoemulsion Droplet Size and Zeta Potential

It has been demonstrated that the physicochemical properties of nanoemulsions such as particle size, dispersion, shape, and also the surface charge are key factors in the release of the core material, the rheological characteristics of colloidal systems, and the cellular uptake. The absorption of nanoparticles by microbial membrane is reported as a two‐step process of binding onto the cell wall and then internalization (Ciani et al. 2007). The attachment of nanoparticles to the cell membrane considerably depends on the surface charge of the emulsion particles (Patil et al. 2007). In the present work, the nanoemulsion droplets were found to have an average diameter of 75 nm, with encapsulation efficiencies of 81%, 73%, and 65% for 1%, 2.5%, and 5% EO nanoemulsion in chitosan, respectively, a zeta potential of −32 mV, and a particle dispersion index of 0.281. No signs of phase separation, sedimentation, or creaming were observed over 12 days of storage at 4°C ± 1°C, indicating high physical stability. The zeta potential is considered a measure of the electrostatic or charge repulsion/attraction between the particles, and also a determining factor in dispersion, aggregation, or flocculation phenomena (Dickinson 2009). The results of a research by Patila et al. (2007) showed that a higher negative zeta potential of a nanoemulsion leads to higher cellular absorption in comparison with nanosystems with a lower negative charge or the ones with a positive surface charge (Patil et al. 2007). Also, our findings are supported by recent studies that confirm nanoemulsions with high negative zeta potential and low PDI exhibit prolonged stability and sustained release characteristics during storage (Acevedo‐Fani et al. 2017; Garcia et al. 2022). It appears that the zeta potential of the nanoemulsion particles in our work (−32 mV) resulted in significant penetration of EO into the bacterial cells and expressed antimicrobial activities.

### 3.4 Chemical Evaluation Results

#### 3.4.1 pH

**FIGURE 1:**
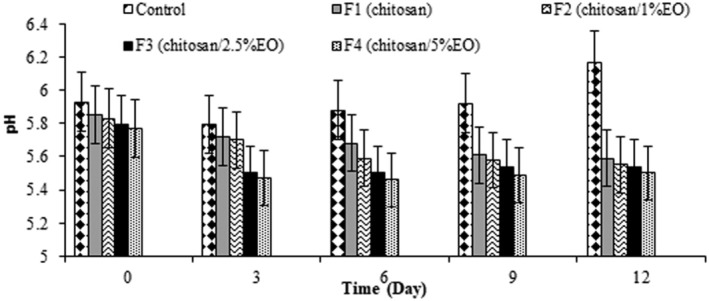
pH values of the ostrich meat samples coated with chitosan and nanoemulsion of *Eryngium campestre* essential oil during storage in the refrigerator.

Significant differences were found between the control and treatments containing pure chitosan (F1) or treatments of chitosan/EO nanoemulsion (F2–F4) (*p* < 0.05) (Figure 1). F4 (Ch + 5%EO) showed the lowest pH value during the storage as reported in Figure 1 (*p* > 0.05). In all the treatments, there was a slight decrease in pH value toward the end of the storage time, while an increase in pH was observed in control samples (without edible coating). The decrease in pH of treatments, especially those containing a higher amount of EO, is due to the presence of organic compounds such as organic acids and the conversion of glycogen in the muscle to lactic acid in case of oxygen supply. Also, during storage in the refrigerator, a slight decrease in pH may occur due to the formation of carbonic acid from the CO_2_ resulting from the metabolism of spoilage‐causing microorganisms (Chmiel et al. 2018).

Similar results were found by Pirnia et al. (2022) that ostrich meat coated by gelatin containing *Hyssopus Officinalis* extract and ascorbic acid showed lower pH than control during the 12‐day storage period. They claimed that gelatin and ascorbic acid appeared to be responsible for controlling the pH of ostrich meat during storage (Pirnia et al. 2022). In the present work, the pH of the treated samples decreased slightly during cold storage, while Fazlara et al. (2017) announced a slight increase in pH values for ostrich meat samples coated with gelatin, but gelatin containing zataria EO caused pH decrease, which shows pure biopolymers do not have the potential of controlling pH. Increasing pH in control during the storage period might result from the degradation of the proteins and other nitrogenous compounds by microorganisms and endogenous enzymes and releasing volatile bases (Alparslan et al. 2016).

#### 3.4.2 Free Fatty Acid Content

**FIGURE 2:**
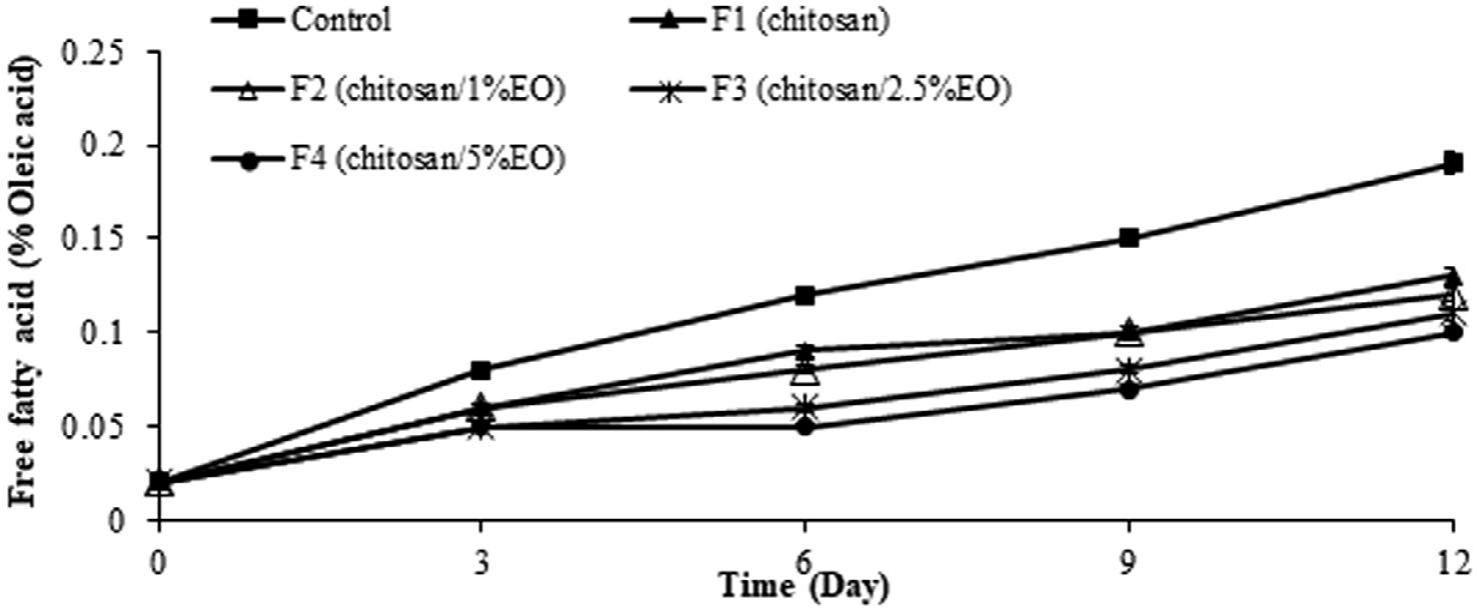
Free fatty acid content of the ostrich meat samples coated with chitosan and nanoemulsion of *Eryngium campestre* essential oil during storage in the refrigerator.

Releasing of FFAs is the result of enzymatic or microbial degradation of triglycerides and phospholipids (de Abreu et al. 2011). According to the results in Figure 2, significant differences were found between the samples coated with chitosan containing 2.5% and 5% nanoemulsion of *E. campestre* EO and the samples coated with chitosan and also the control (*p* < 0.05). FFA content showed a slow increase in treated samples during the storage period. This increase was significantly higher in the control. The meat pieces coated with chitosan/2.5% and 5% EO nanoemulsion had lower FFA content (0.1%–0.11%) compared to the samples coated with pure chitosan (0.13%) (*p* < 0.05). As it is obvious from the results, composite edible coatings of chitosan/5% EO nanoemulsion caused the lowest FFA release in the samples (*p* < 0.05) followed by chitosan/2.5% EO nanoemulsion (*p* > 0.05). It was found that the addition of *E. campestre* EO nanoemulsion to chitosan edible coating created a synergistic antioxidant effect, as reported in previous studies (Ghafari et al. 2024). The FFA contents in the samples during storage were as follows: Control > chitosan > chitosan/1% EO nanoemulsion > chitosan/2.5% EO nanoemulsion > chitosan/5% EO nanoemulsion.

In a study by Diniz do Nasciment et al. (2020), germacrene D (the main component of *E. campestre* EO in this work) has been studied for its potential effects on the inhibition of FFA release in meat. Their results suggested that germacrene D might play an important role in modulating lipid metabolism through influencing the enzymes responsible for FFA release, thereby leading to reduced lipid oxidation and preserving meat chemical and organoleptic quality (Diniz do Nascimento et al. 2020).

#### 3.4.3 Total Volatile Base Nitrogen (TVB‐N)

**FIGURE 3:**
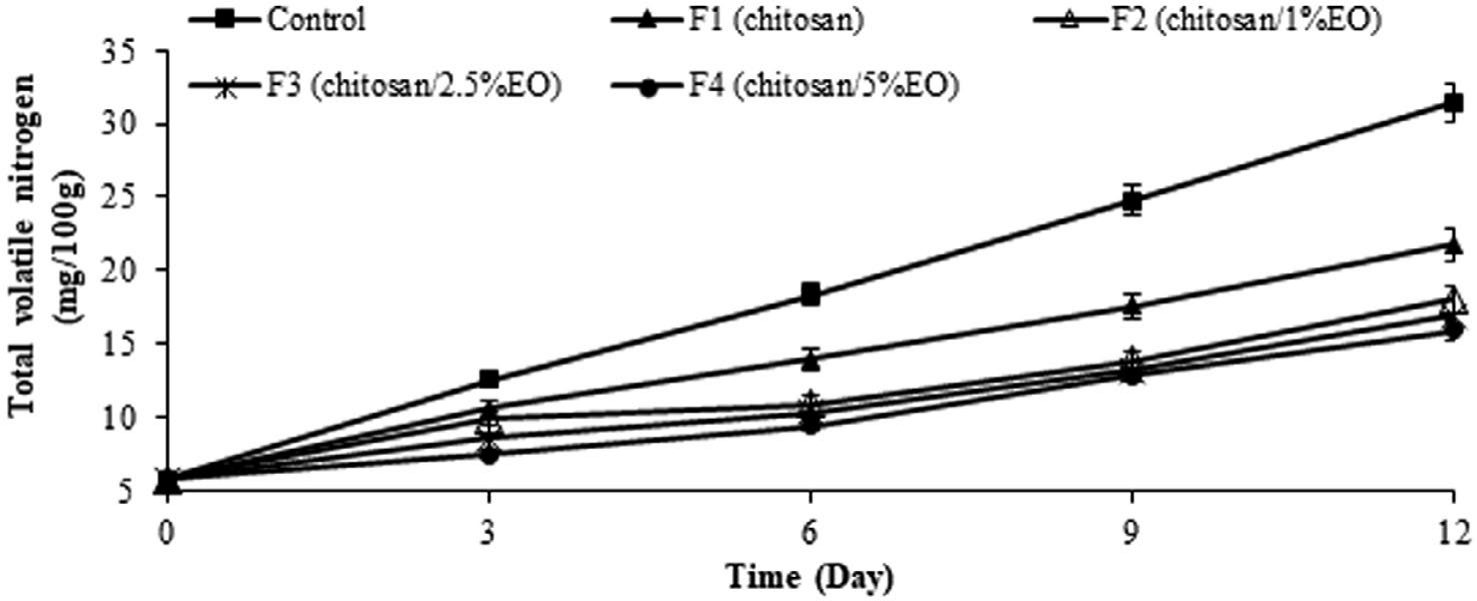
TVB‐N content of the ostrich meat samples coated with chitosan and nanoemulsion of *Eryngium campestre* essential oil during storage in the refrigerator.

The total volatile base nitrogen in meat products is measured as a determining factor of freshness. During storage, the TVB‐N value increases due to the activities of spoilage microorganisms and also the endogenous enzymes. The results obtained for TVB‐N in ostrich meat samples (Figure 3) showed that TVB‐N increased during 12‐day storage in the control group and reached the value of 31.45 mg/100 g on Day 12. In F1 (pure chitosan), this value reached 21.79 mg/100 g at Day 12, followed by F2 (18.07 mg/100 g), F3 (16.93 mg/100 g), and F4 (15.9 mg/100 g) (*p* < 0.05). As seen, the lowest TVB‐N values belonged to F4 (chitosan/5% EO nanoemulsion) during the cold storage (*p* < 0.05). Although the difference between F4 and F3 was statistically significant (*p* < 0.05), the practical reduction in TVB‐N (only 1.03 mg/100 g) is relatively small. Therefore, while F4 demonstrated slightly improved preservation, the benefit of using a higher EO concentration should be weighed against cost‐effectiveness, and further optimization is needed to determine the most efficient formulation.

In all chitosan edible coating groups containing *E. campestre* EO nanoemulsion, TVB‐N was significantly lower than the control and groups of chitosan without EO nanoemulsion (*p* < 0.05) throughout the storage period. In a study by Fazlara et al. (2017) on applying gelatin edible coating with zataria EO for ostrich meat, pure gelatin edible coating had no significant inhibitory effect on TVB‐N increase. They found that gelatin combined with 1.5% zataria EO had a considerable inhibitory activity against TVB‐N release, which was due to the presence of polyphenolic and terpenoid compounds that reduced the bacterial population. Farhadi et al. (2022) investigated the effect of edible chitosan containing *Froriepia subpinnata* extract during the storage of Nile tilapia fillets. They reported that coated samples with chitosan and 2% of *F*. *subpinnata* extract had the lowest TVB‐N value and better quality than the control group in terms of investigated microbial and chemical indexes (Farhadi et al. 2022). Research suggests that *Eryngium campestre* , with its natural potential antioxidant and antimicrobial activities, may reduce the release of total volatile nitrogen compounds, thereby slowing down the spoilage process in meat products. The plant's bioactive compounds, flavonoids, and phenolic acids particularly interact with bacterial enzymes and reduce the production of volatile nitrogenous and basic compounds (Soumia 2018).

#### 3.4.4 Peroxide Value

**FIGURE 4:**
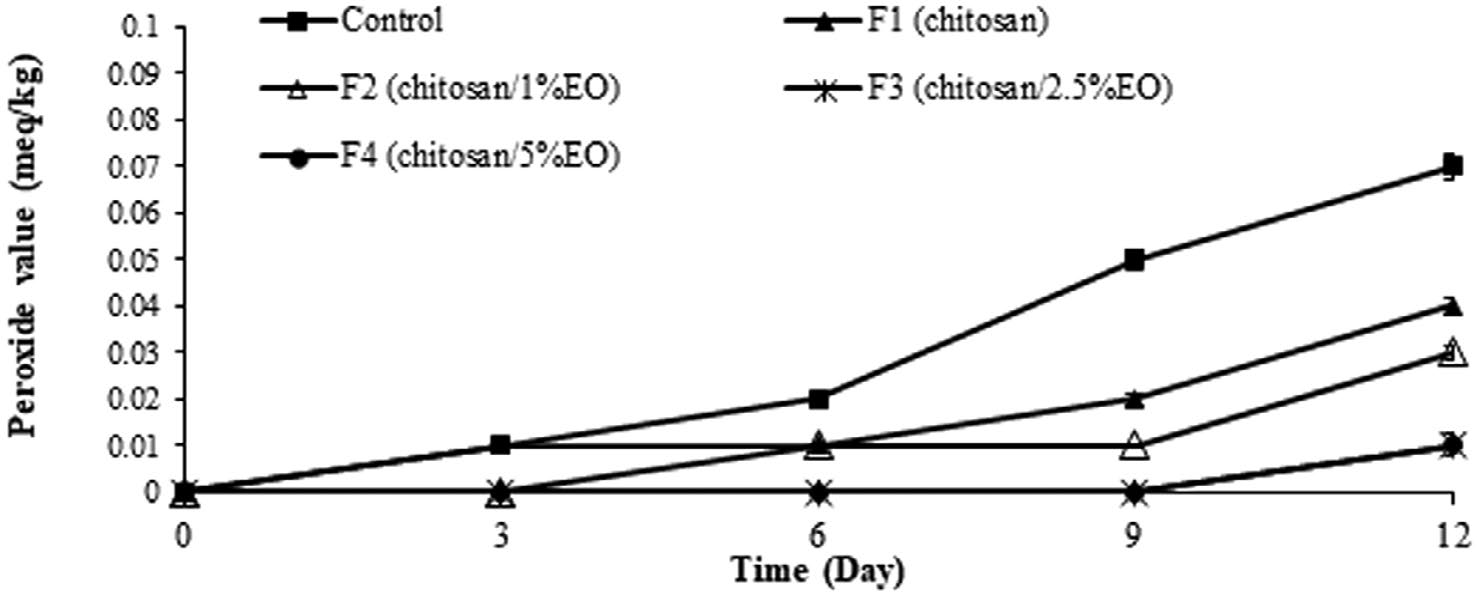
Peroxide value of the ostrich meat samples coated with chitosan and nanoemulsion of *Eryngium campestre* essential oil during storage in the refrigerator.

Peroxide value is an indicator of hydroperoxides' presence as the primary products of lipids oxidation and their degradation leads to the formation of a wide range of hydrocarbons, carbonyl compounds, furans, ketones, and other products that create the rancid odor and taste in the food product. Oxidative deterioration progress significantly in the control samples during storage (*p* < 0.05) and the treated samples with chitosan (F1) and chitosan/EO nanoemulsion (F2, F3, and F4) showed lower PV compared to control (Figure 4) (*p* < 0.05). There was no significant difference between the PV of the treated samples (*p* > 0.05). This may suggest that chitosan alone exerted strong inherent antioxidant activity, potentially masking any additional effect of the EO. Moreover, it is possible that the EO's contribution to peroxide inhibition was more pronounced at later oxidative stages or that its active compounds were partially degraded or not fully released during the study period, limiting their impact on PV levels. Jonaidi Jafari et al. (2018) studied the effect of chitosan edible coating containing the ethanolic extract of propolis on the quality of chicken fillets stored at 4°C. They announced that oxidative spoilage in the treated samples and control was not significantly different during the first 3 days, but on Days 6 and 9, the peroxide value of the samples treated with chitosan/propolis extract was found lower than control and pure chitosan (Jonaidi Jafari et al. 2018); the same results were obtained in the present study, and samples containing chitosan/EO nanoemulsion had lower PV. Researchers attributed this result to the antioxidant activity of EO compounds, especially polyphenol contents and terpenes (Heydari et al. 2015) like germacrene, campestrolide, salvial, and α‐bisabolol in our study. It is demonstrated that phenolic antioxidants inhibit the formation of FFA radicals, which react with oxygen or absorb it in the auto‐oxidation process; therefore, they delay the onset of the auto‐oxidation in fats (Abdollahi et al. 2014).

#### 3.4.5 Thiobarbituric Acid Reactive Substances

**FIGURE 5:**
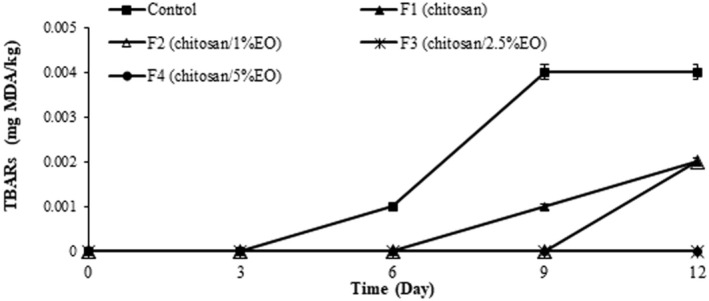
TBARs of the ostrich meat samples coated with chitosan and nanoemulsion of *Eryngium campestre* essential oil during storage in the refrigerator.

Releasing TBA reactive substances was significantly higher in the control (*p* < 0.05) and the samples coated with chitosan/2.5 and 5% EO nanoemulsion (F3 and F4) showed no detectable TBARs during storage (Figure 5) (*p* < 0.05). At the end of the storage period, there was no significant difference between the TBARs in the samples treated with chitosan (F1) and chitosan/1% EO nanoemulsion (F2) (*p* > 0.05). The significantly higher TBARs levels in the control group confirm the progression of lipid oxidation in uncoated ostrich meat during storage, consistent with previous studies highlighting the pro‐oxidative nature of refrigerated conditions (Yan et al. 2024). In contrast, the absence of detectable TBARs in F3 and F4 suggests that the combination of chitosan with *E. campestre* EO nanoemulsions at higher concentrations effectively inhibited secondary lipid oxidation, likely due to their strong antioxidant properties. The comparable TBARs values between F1 and F2 indicate that 1% EO may be insufficient to enhance the antioxidative action of chitosan alone (Muñoz‐Tebar et al. 2023). These findings demonstrate the synergistic effect of biopolymer coatings and EO nanoemulsions in preserving meat quality.

### 3.5 Microbiological Evaluation

#### 3.5.1 Mesophilic Bacteria Count

The chemical composition of ostrich meat makes it a suitable media for bacterial growth and spoilage during storage time. The number of mesophilic bacteria in the control group increased during storage and its population reached 8.34 log cfu/g on Day 12 (Table 3). The mesophilic count of coated samples incorporated with chitosan and chitosan/nanoemulsion (F1‐F4) was lower compared to control (*p* < 0.05). Also, samples coated with chitosan/nanoemulsion (F2‐F4) showed a lower count in comparison to pure chitosan‐coated samples (*p* < 0.05). In the F4 (chitosan/5% nanoemulsion) group, the count was 1.27 log cfu/g on Day 12 which showed a significant difference compared to the other treatments (*p* < 0.05). As seen in Table 3, the total population of mesophilic bacteria was as follows: control > F1 > F2 > F3 > F4. Coating the ostrich meat samples with *E. campestre* EO nanoemulsion exerted a higher inhibitory effect against bacterial growth compared to pure chitosan edible coating (*p* < 0.05). It seems that there is a synergistic effect between biopolymers like chitosan and EO components in the inhibition of bacterial growth. The same considerable decrease was reported by Heydari et al. (2015) in the bacterial count of bighead carp fillets coated with sodium alginate enriched with *Mentha longifolia* EO during 12‐day storage at 4°C (Heydari et al. 2015). Gao et al. (2024) reviewed the effect of *E. campestre* EO incorporated in several types of gums on the shelf life of meat and chicken fillets during storage at refrigerated temperature. The results showed that *E. campestre* extract significantly improved the antibacterial activity of the guar edible coating (Gao et al. 2024). Langroodi et al. (2018) concluded that the application of an edible coating supplemented with herbal EOs and extracts could possibly induce the structural destruction of mitochondrial and cellular membrane lipids in the bacteria and prohibit microbial proliferation (Langroodi et al. 2018). This effect is attributed to the presence of the phenolic compounds and terpenoids that possess antimicrobial activities. As declared by previous studies, biopolymers act as a barrier against oxygen transfer which results in the growth inhibition of aerobic bacteria (Song et al. 2011).

**TABLE 3:** Changes of mesophilic count (log cfu/g) in ostrich meat coated with chitosan containing nanoemulsion of *Eryngium campestre* essential oil during storage at 4°C ± 1°C.

	Time (day)				
	0	3	6	9	12
Control	2.55 ± 0.09^Aa*^	3.95 ± 0.15^Ba^	5.65 ± 0.20^Ca^	6.48 ± 0.11^Da^	8.34 ± 0.05^Ea^
Chitosan	2.55 ± 0.09^Aa^	2.90 ± 0.85^Bb^	3.77 ± 0.19^Cb^	3.81 ± 0.35^Cb^	4.09 ± 0.04^Db^
Chitosan +1% EO	2.55 ± 0.09^Aa^	2.75 ± 0.33^Bb^	2.51 ± 0.50^Cc^	2.53 ± 0.29^Cc^	2.88 ± 0.25^Bc^
Chitosan +2.5% EO	2.55 ± 0.09^Aa^	2.17 ± 0.49^Bc^	2.01 ± 0.19^Bd^	1.94 ± 0.30^Bd^	1.93 ± 0.34^Bd^
Chitosan +5% EO	2.55 ± 0.09^Aa^	1.53 ± 0.23^Bd^	1.36 ± 0.51^Ce^	1.31 ± 0.40^Ce^	1.27 ± 0.10^Ce^

*Note:* ^a‐e^Represents a statistically significant difference (*p* < 0.05) between means of the treatments at the same day (in each column). ^A‐E^Represents a statistically significant difference of means for the same treatment during the storage period (in each row). *Data are presented as mean ± SD.

#### 3.5.2 Psychrotrophic Bacteria

At cold temperatures, Gram‐negative psychrotrophics are considered the main group of microorganisms responsible for the spoilage of meat (Saenz‐García et al. 2020). As presented in Table 4, on Days 3, 6, and 9, the psychrotrophic count of samples treated with chitosan incorporated with 2.5% and 5% EO nanoemulsion was about 2.5–3 log cfu/g lower than the samples treated with chitosan or chitosan/1% EO nanoemulsion and about 5–5.5 log cfu/g lower than control. The highest antibacterial activity of F3 and F4 was obtained at day 12 when the psychrotrophics count was 5–6.5 log cfu/g, indicating that F3 and F4 were effective against the growth of psychrotrophic bacteria (*p* < 0.05). Also, a significant difference was observed between the psychrotrophic population of F1 (chitosan treated samples) and control (*p* < 0.05), which indicated the inhibitory effect of pure chitosan against the growth of this group of bacteria. Langroodi et al. (2018) evaluated the preservative effects of sumac hydro‐alcoholic extract and chitosan coating enriched with *Zataria multiflora* Boiss EO on the quality of beef during cold storage. In all treatments, the population of psychrotrophic bacteria significantly decreased compared to the control group at the end of storage time. They claimed that the use of EOs and extracts in combination with chitosan might have a synergistic antimicrobial effect (Langroodi et al. 2018). In a study by Ghafari et al. (2024), the effect of nanochitosan‐Iranian tragacanth gum composite film along with 1% *E. campestre* EO on the shelf life of goat meat was investigated, and a considerable decrease in the growth rate of psychrotrophics was found (Ghafari et al. 2024). Also, Zamani Faradonbeh et al. (2024) reported a significant decrease in psychrotrophic bacteria in ostrich meat slices coated with *Ocimum basilicum* seed mucilage infused with *Hypericum perforatum* extract (Faradonbeh et al. 2024). Our results are in agreement with the above studies that composing chitosan with EOs or extracts leads to the enhancement of the antimicrobial effect of both of them.

**TABLE 4:** Changes of psychrotrophic bacteria count (log cfu/g) in ostrich meat coated with chitosan containing a nanoemulsion of *Eryngium campestre* essential oil during storage at 4°C ± 1°C.

	Time (day)				
	0	3	6	9	12
Control	2.18 ± 0.05^Aa*^	4.15 ± 0.20^Ba^	5.19 ± 0.05^Ca^	6.41 ± 0.19^Da^	7.31 ± 0.55^Ea^
Chitosan	2.18 ± 0.05^Aa^	2.95 ± 0.32^Bb^	3.39 ± 0.41^Cb^	4.25 ± 0.10^Db^	5.20 ± 0.16^Eb^
Chitosan +1% EO	2.18 ± 0.05^Aa^	2.30 ± 0.25 ^Bc^	2.76 ± 0.09^Cc^	3.02 ± 0.21^Dc^	3.39 ± 0.45^Ec^
Chitosan +2.5% EO	2.18 ± 0.05^Aa^	1.92 ± 0.38^Bd^	1.71 ± 0.00^Cd^	1.54 ± 0.20^Dd^	1.37 ± 0.06^Dd^
Chitosan +5% EO	2.18 ± 0.05^Aa^	1.88 ± 0.47^Bd^	1.55 ± 0.70^Cd^	1.15 ± 0.37^De^	0.93 ± 0.30^Ee^

*Note:* ^a‐e^represents a statistically significant difference (*p* < 0.05) between means of the treatments on the same day (in each column). ^A‐E^represents a statistically significant difference of means for the same treatment during the storage period (in each row). *Data are presented as mean ± SD.

#### 3.5.3 Enterobacteriaceae Count

According to the data in Table 5, the control samples exhibited the highest population of Enterobacteriaceae during storage at 4°C, surpassing the commonly accepted safety limit of 7 log CFU/g for poultry meat (*p* < 0.05). All edible coating treatments significantly inhibited *Enterobacteriaceae* growth compared to the control (*p* < 0.05), following the order: F4 > F3 > F2 > F1. Among these, F3 (2.5% EO) and F4 (5% EO) showed the strongest antibacterial activity, with no significant difference between them (*p* > 0.05), suggesting that the 2.5% EO concentration may be sufficient to exert maximal antimicrobial effects, potentially rendering the 5% EO unnecessary. Similar inhibitory effects of chitosan and zataria EO combinations against *Enterobacteriaceae* have been reported by Langroodi et al. (2018) in raw beef (Langroodi et al. 2018). The potent antibacterial action is likely attributed to bioactive compounds such as flavonoids, sesquiterpenes (e.g., germacrene D), and terpenoids present in *E. campestre* EO, known for their cytostatic, antimicrobial, and antioxidant properties (Kademi and Garba 2017). Overall, the chitosan/EO nanoemulsion coatings demonstrated strong potential to control microbial growth and enhance meat safety.

**TABLE 5:** Changes of *Enterobacteriaceae* count (log cfu/g) in ostrich meat coated with chitosan containing nanoemulsion of *Eryngium campestre* essential oil during storage at 4°C ± 1°C.

	Time (day)				
	0	3	6	9	12
Control	1.25 ± 0.07^Aa*^	2.51 ± 0.10^Ba^	3.34 ± 0.05^Ca^	4.87 ± 0.10^Da^	5.45 ± 0.10^Ea^
Chitosan	1.25 ± 0.07^Aa^	2.18 ± 0.15^Bb^	2.49 ± 0.21^Cb^	3.50 ± 0.05^Db^	4.17 ± 0.11^Eb^
Chitosan +1% EO	1.25 ± 0.07^Aa^	1.55 ± 0.35^Bc^	1.88 ± 0.15^Cc^	2.16 ± 0.30^Dc^	2.75 ± 0.20^Ec^
Chitosan +2.5% EO	1.25 ± 0.07^Aa^	1.17 ± 0.10^Ad^	1.10 ± 0.35^Ad^	1.03 ± 0.10^Bd^	0.85 ± 0.10^Bd^
Chitosan +5% EO	1.25 ± 0.07^Aa^	1.05 ± 0.15^Ad^	0.91 ± 0.01^Bd^	0.85 ± 0.07^Bd^	0.71 ± 0.00^Cd^

*Note:* ^a‐e^Represents a statistically significant difference (*p* < 0.05) between means of the treatments on the same day (in each column). ^A‐E^Represents a statistically significant difference of means for the same treatment during the storage period (in each row). *Data are presented as mean ± SD.

#### 3.5.4 Lactic Acid Bacteria (LAB) Count

LAB are significant contributors to ostrich meat spoilage due to their ability to ferment carbohydrates and produce lactic acid. They cause off‐flavors, texture changes, and a decline in meat quality, especially in low‐oxygen environments like vacuum‐packed or refrigerated meat. LAB's metabolic byproducts, such as organic acids, lead to sour taste and spoilage (Carneiro et al. 2024). Alterations in the LAB population in ostrich meat samples treated with chitosan edible coating and varying concentrations of *E. campestre* EO nanoemulsion during storage at 4°C ± 1°C are presented in Table 6. Initially, the LAB count was around 3.88 log cfu/g, progressively increasing during storage, reaching approximately 5–7 log cfu/g and 7 log cfu/g by Day 12 for the coated samples and control, respectively. We observed that pure chitosan coatings inhibited LAB growth, while the incorporation of *E. campestre* EO nanoemulsion resulted in a greater reduction in LAB count (*p* < 0.05). The growth rate of LAB in samples treated with formulations F3 and F4 was the lowest, and there was no significant difference between the inhibitory effects of F3 and F4 (*p* > 0.05). As indicated in Table 6, LAB populations decreased from Days 1 to 6, but from Days 9 to 12, a noticeable increase in LAB growth was observed. This biphasic pattern (where LAB counts initially decreased and later rebounded) can be explained by the antimicrobial effects of chitosan and the EO nanoemulsion, which likely suppressed LAB growth in the early stages. Over time, LAB strains, such as lactobacilli, can adapt to sublethal concentrations of antimicrobial agents, employing mechanisms such as biofilm formation, membrane composition changes, efflux pumps, or specific enzymatic pathways. However, the decrease in LAB counts from Days 1 to 6 did not correlate directly with a continued decrease in pH. The observed decrease in pH during this period is expected, as LAB produce lactic acid. Yet, the rebound of LAB populations from Days 9 to 12 did not correspond to a further decrease in pH, which might seem inconsistent. This discrepancy can be attributed to several factors like other microbial activities and the buffering capacity of the meat matrix (Diniz‐Silva et al. 2020; Yao et al. 2025).

**TABLE 6:** Changes of Lactic acid bacteria count (log cfu/g) in ostrich meat coated with chitosan containing nanoemulsion of *Eryngium campestre* essential oil during storage at 4°C ± 1°C.

	Time (day)				
	0	3	6	9	12
Control	3.85 ± 0.20^Aa*^	5.10 ± 0.05^Ba^	5.95 ± 0.10^Ca^	6.55 ± 0.03^Da^	7.01 ± 0.30^Ea^
Chitosan	3.85 ± 0.20^Aa^	4.63 ± 0.01^Bb^	5.25 ± 0.38^Cb^	5.81 ± 0.25^Db^	6.19 ± 0.05^Eb^
chitosan +1% EO	3.85 ± 0.20^Aa^	4.51 ± 0.26^Bb^	4.88 ± 0.10^Cc^	5.75 ± 0.22^Cb^	6.15 ± 0.51^Db^
chitosan +2.5% EO	3.85 ± 0.20^Aa^	4.40 ± 0.30^Bb^	4.71 ± 0.04^Cc^	5.13 ± 0.10^Dc^	5.79 ± 0.29^Ec^
chitosan +5% EO	3.85 ± 0.20^Aa^	4.25 ± 0.10^Bc^	4.39 ± 0.07^Bd^	4.91 ± 0.33^Cd^	5.08 ± 0.05^Cd^

*Note:* ^a‐e^Represents a statistically significant difference (*p* < 0.05) between means of the treatments on the same day (in each column). ^A‐E^Represents a statistically significant difference of means for the same treatment during the storage period (in each row). *Data are presented as mean ± SD.

### 3.6 Drip Loss

**FIGURE 6:**
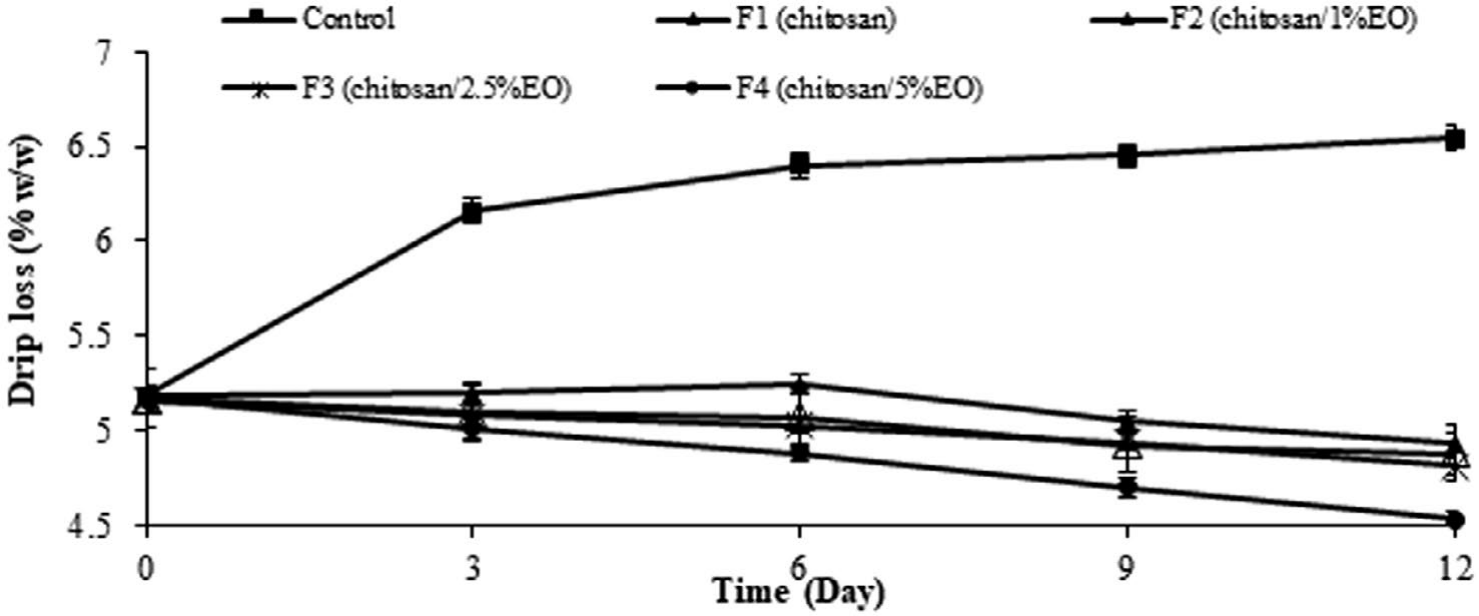
Drip loss percentage of ostrich meat coated with chitosan containing nanoemulsion of *Eryngium campestre* essential oil during storage at 4°C ± 1°C.

The initial drip loss of the fresh ostrich meat samples was approximately 5.17% (Figure 6). Drip loss decreased in all samples except for an increase in control during the storage time (*p* < 0.05). Samples coated with chitosan/2.5% EO nanoemulsion and chitosan/5% EO nanoemulsion had the lowest drip loss during the storage period (*p* < 0.05). The drip loss of the samples coated with pure chitosan or chitosan/EO nanoemulsion was significantly lower than the control (without edible coating) (*p* < 0.05). Also, the drip loss in coated samples on 12th day of storage was significantly lower than those on the 3rd, 6th, and 9th day of refrigerated storage (*p* < 0.05). It seems chitosan exerted a considerable effect on inhibiting moisture loss from the texture. As indicated by Zheng et al. (2023), the drip loss percentage of the fresh meat samples coated with chitosan incorporated with oregano EO was significantly lower than the control (Zheng et al. 2023) which is similar to the findings of the present study. Also, Gaba et al. (2022) reported the significant effect of chitosan‐based edible coatings incorporated with thyme EO on the quality of beef meat (Gaba et al. 2022). In this work, the ostrich meat samples treated with chitosan and EO nanoemulsion reflected significantly lower drip losses as compared to control that demonstrated higher muscle integrity and water holding capacity of the texture (Yang et al. 2018). Degradation of myofibrillar proteins and collagens as the result of microbial activity on the meat surface leads to the release of intercellular fluids and drip loss during storage (Roslan et al. 2019). The low drip loss shows lower microbial spoilage in coated samples which is proven by Pabast et al. (2018) who achieved decreasing the drip loss through developing chitosan‐based edible coatings enriched with EOs in lamb meat. It is established by previous studies that EOs combined with chitosan enhance the edible coating's effectiveness in preventing moisture loss and spoilage, this is important because drip loss can lead to reduced meat quality, affecting texture and flavor. Moreover, the antimicrobial activity of the EOs helps inhibit microbial growth, further improving meat shelf life. Therefore, chitosan/EO edible coatings can significantly reduce the drip loss while maintaining the meat's quality (Pabast et al. 2018).

### 3.7 Texture

**FIGURE 7:**
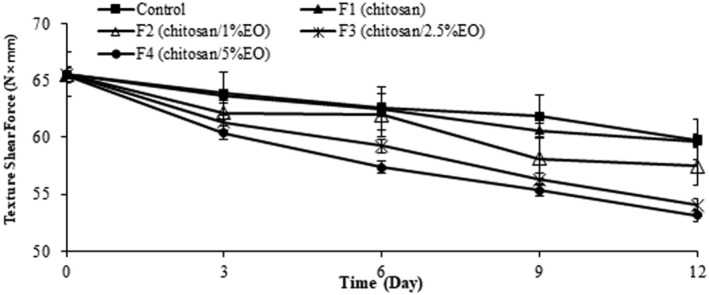
Texture shear force of ostrich meat coated with chitosan containing nanoemulsion of *Eryngium campestre* essential oil during storage at 4°C ± 1°C.

In food texture analysis, shear force is used to assess the tenderness of foods like meat. It measures the amount of force needed to break or deform the food, typically through a texture analyzer. A higher shear force indicates tougher or firmer food, while a lower shear force suggests that the food is softer or more tender (Mabrouki et al. 2024). The mean texture shear force of the samples on Day 0 was 65.5 N*mm, which gradually decreased in all samples during cold storage (*p* < 0.05) (Figure 7). The ostrich meat samples coated with F2, F3, and F4 showed significantly lower (*p* < 0.05) firmness on Days 6, 9, and 12 of storage compared to the control and F1. It is found that chitosan edible coatings incorporated with EO provided higher softness (*p* < 0.05) in the samples in comparison to edible coatings without EO, as seen in Figure 7. The lowest shear force belongs to F3 and F4, indicating that EO nanoemulsion significantly decreased the texture firmness and increased the tenderness, as confirmed by the sensory scores of tenderness presented in Table 7. Garavito et al. (2020) reported that the firmness of the chicken breast fillets coated with biopolymers containing oregano EO decreased throughout the storage time, and this value was lower for the coated fillets than for the control (Garavito et al. 2020). This can be due to a higher rate of degradation of muscle fibers in the coated fillets as the result of the greater amount of moisture retained by the edible coatings, which enhanced the development of collagen hydrolysis and protein degradation (Mir et al. 2017).

**TABLE 7:** Sensory scores of ostrich meat coated with chitosan containing nanoemulsion of *Eryngium campestre* essential oil during storage at 4°C ± 1°C.

	Time (day)				
	0	3	6	9	12
Odor					
Control	8.0 ± 0.00^Aa*^	8.0 ± 0.01^Aa^	8.0 ± 0.0^Ab^	7.0 ± 0.00^Ba^	5.5 ± 0.02^Ca^
Chitosan	8.0 ± 0.02^Aa^	8.0 ± 0.01^Aa^	7.0 ± 0.00^Ba^	7.0 ± 0.03^Ba^	6.0 ± 0.00^Cb^
Chitosan +1% EO	8.5 ± 0.00^Ab^	8.5 ± 0.00^Ab^	8.0 ± 0.01^Bb^	7.5 ± 0.02^Cb^	6.5 ± 0.01^Dc^
Chitosan +2.5% EO	9.0 ± 0.00^Ac^	9.0 ± 0.00^Ac^	8.5 ± 0.01^Bc^	8.0 ± 0.00^Cc^	7.0 ± 0.00^Dd^
Chitosan +5% EO	9.0 ± 0.00^Ac^	9.0 ± 0.00^Ac^	9.0 ± 0.00^Ad^	8.5 ± 0.00^Bd^	8.0 ± 0.01^Ce^
Flavor					
Control	8.0 ± 0.00^Aa^	8.0 ± 0.00^Aa^	7.5 ± 0.01^Ba^	6.5 ± 0.00^Ca^	6.5 ± 0.01^Ca^
Chitosan	8.0 ± 0.01^Aa^	8.0 ± 0.02^Aa^	8.0 ± 0.00^Ab^	7.0 ± 0.03^Bb^	6.5 ± 0.00^Ca^
Chitosan +1% EO	8.5 ± 0.00^Ab^	8.5 ± 0.00^Ab^	8.5 ± 0.00^Ac^	7.5 ± 0.01^Bc^	7.0 ± 0.01^Cb^
Chitosan +2.5% EO	8.5 ± 0.00^Ab^	9.0 ± 0.00^Bc^	9.0 ± 0.01^Cd^	8.5 ± 0.00^Ad^	8.0 ± 0.00^Cc^
Chitosan +5% EO	8.5 ± 0.00^Ab^	9.0 ± 0.00^Bc^	9.0 ± 0.00^Bd^	8.5 ± 0.00^Ad^	8.0 ± 0.00^Cc^
Color					
Control	9.0 ± 0.01^Aa^	8.0 ± 0.00^Ba^	7.5 ± 0.02^Ca^	6.5 ± 0.00^Da^	6.5 ± 0.01^Da^
Chitosan	9.0 ± 0.00^Aa^	8.5 ± 0.02^Bb^	8.0 ± 0.00^Cb^	7.0 ± 0.03^Db^	7.0 ± 0.00^Db^
Chitosan +1% EO	9.0 ± 0.00^Aa^	8.5 ± 0.00^Bb^	8.0 ± 0.00^Cc^	7.0 ± 0.00^Db^	7.0 ± 0.00^Db^
Chitosan +2.5% EO	9.0 ± 0.00^Aa^	9.0 ± 0.00^Ac^	8.5 ± 0.02^Bd^	8.5 ± 0.00^Bc^	8.0 ± 0.00^Cc^
Chitosan +5% EO	9.0 ± 0.00^Aa^	9.0 ± 0.00^Ac^	8.5 ± 0.00^Bd^	8.5 ± 0.00^Bc^	8.0 ± 0.01^Cc^
Tenderness					
Control	5.0 ± 0.00^Aa^	6.0 ± 0.00^Ba^	6.0 ± 0.02^Ba^	6.0 ± 0.00^Ba^	7.0 ± 0.01^Ca^
Chitosan	5.0 ± 0.00^Aa^	6.0 ± 0.02^Ba^	6.0 ± 0.00^Ba^	7.0 ± 0.03^Cb^	7.5 ± 0.00^Db^
Chitosan +1% EO	5.0 ± 0.00^Aa^	6.0 ± 0.00^Ba^	6.5 ± 0.00^Cb^	7.0 ± 0.00^Db^	8.0 ± 0.00^Ec^
Chitosan +2.5% EO	5.5 ± 0.01^Ab^	6.5 ± 0.02^Bb^	7.0 ± 0.02^Cc^	8.0 ± 0.00^Dc^	8.5 ± 0.00^Ed^
Chitosan +5% EO	6.0 ± 0.00^Ac^	7.0 ± 0.00^Bc^	8.0 ± 0.00^Cd^	8.5 ± 0.01^Dd^	9.0 ± 0.01^Ee^
Overall acceptability					
Control	7.5 ± 0.00^Aa^	7.0 ± 0.00^Ba^	6.0 ± 0.02^Ca^	5.5 ± 0.00^Da^	5.0 ± 0.00^Ea^
Chitosan	7.5 ± 0.00^Aa^	7.0 ± 0.02^Ba^	7.0 ± 0.00^Bb^	6.5 ± 0.00^Cb^	5.0 ± 0.00^Da^
Chitosan +1% EO	8.0 ± 0.00^Ab^	7.0 ± 0.00^Ba^	7.0 ± 0.00^Bb^	6.5 ± 0.00^Cb^	5.5 ± 0.00^Db^
Chitosan +2.5% EO	8.0 ± 0.00^Ab^	7.5 ± 0.00^Bb^	7.5 ± 0.02^Bc^	6.5 ± 0.00^Cb^	6.0 ± 0.00^Dc^
Chitosan +5% EO	8.5 ± 0.00^Ac^	8.0 ± 0.00^Bc^	8.5 ± 0.00^Ad^	7.0 ± 0.00^Cc^	7.0 ± 0.01^Cd^

*Note:* ^a‐e^Represents a statistically significant difference (*p* < 0.05) between means of the treatments on the same day (in each column). ^A‐E^Represents a statistically significant difference of means for the same treatment during the storage period (in each row). *Data are presented as mean ± SD.

### 3.8 Sensory Scores

Sensory evaluation of ostrich meat treated with chitosan containing nanoemulsion of *E. campestre* EO presented varied findings throughout 12‐day storage period at 4°C ± 1°C (Table 7). Day 0 odor scores in every treatment were high, with the highest score among them recorded by chitosan/5% EO, which was as high as (9.0 ± 0.00) that is, odor with highly pleasing attributes as opposed to that of the control (8.0 ± 0.00). Over time, the odor scores decreased for every treatment, with the control decreasing fastest, to 5.5 ± 0.02 on Day 12. In contrast, the chitosan/5% EO treatment had the highest scores (8.0 ± 0.01) by Day 12. This shows that the incorporation of EO into the chitosan coating enhances the freshness of the meat upon storage, perhaps due to the antimicrobial and antioxidant activities of the EO (Ghafari et al. 2024). The same tendencies were found for flavor. The chitosan/5% EO treatment maintained the highest initial flavor score (8.5 ± 0.00) and continued leading in the scores during the storage period, dropping to 8.0 ± 0.00 on Day 12. The control group showed a significant decrease in flavor scores, from 8.0 ± 0.00 on Day 0 to 6.5 ± 0.01 on Day 12, suggesting possible flavor degradation. Enhancement of the retention of flavor by EO‐enriched chitosan coatings is likely caused by the bioactive compounds present in the EO, which have been shown to aid in the stabilization of flavor and in the inhibition of microorganisms (Ghafari et al. 2024). Color scores for all the treatments started high, with control and chitosan groups having a score of 9.0 ± 0.01 on Day 0. The scores for these groups reduced steadily over time, with control reaching 6.5 ± 0.01 on Day 12. The chitosan/5% EO group, however, had the most stable color retention, scoring 8.0 ± 0.01 on Day 12. This suggests that the coating can help maintain the look of the ostrich meat, perhaps by holding back oxidative changes in the pigments of the meat (Aminzare et al. 2019). The control group tenderness scores started at 5.0 ± 0.00 and increased progressively to 7.0 ± 0.01 on Day 12. For the chitosan/5% EO group, however, the maximum scores were 9.0 ± 0.01 on Day 12, which are consistent with the data obtained for texture firmness and shear force (Figure 7). The enhanced tenderness of the chitosan/EO treatments can be attributed to the role of chitosan in enhancing moisture retention, hence avoiding dehydration and maintaining the meat texture (Adam 2021). The overall acceptability scores followed the trends for the other sensory attributes. The chitosan/5% EO group scored the highest across the board, starting at 8.5 ± 0.00 on Day 0 and remaining high at 7.0 ± 0.01 on Day 12. The control group experienced a significant drop in acceptability, with scores decreasing from 7.5 ± 0.00 on Day 0 to 5.0 ± 0.00 on Day 12. The overall higher acceptability of the chitosan/EO treatments can be explained by the combined impacts of improved sensory attributes, including odor, flavor, and tenderness.

The application of chitosan coating, especially with higher concentrations of *E. campestre* EO, clearly enhanced the sensory attributes of ostrich meat during cold storage. The antioxidative and antimicrobial effects of the EO must have been accountable for better odor, flavor, color, and tenderness retention.

## 4 Conclusions

The combined effect of chitosan and nanoemulsion of *E. campestre* EO on the physical, chemical, and microbiological quality of refrigerated ostrich meat was evaluated. This is the first report demonstrating the combined use of chitosan and *E. campestre* EO in nanoemulsified form specifically for ostrich meat preservation. The formulation notably improved antioxidant properties, reduced lipid oxidation, and maintained physicochemical and microbiological quality over time, highlighting its potential as a natural and effective alternative to synthetic preservatives in the meat industry. The results indicated that applying chitosan edible coating incorporated with EO nanoemulsion creates higher preservative activity compared to chitosan alone. The treatments containing chitosan and 2.5% or 5% EO nanoemulsion showed a significant preservative effect on the samples and also exerted preservative effects on texture, organoleptic properties, and drip loss. Samples treated with the mentioned edible coatings above had significantly lower FFA, PV, TVB‐N content, and microbial count during cold storage in comparison to the samples coated with pure chitosan and control. The treatments' efficiency as antimicrobial agents and maintaining physicochemical properties were as follows: chitosan/5% EO nanoemulsion > chitosan/2.5% EO nanoemulsion > chitosan/1% EO nanoemulsion > chitosan. The findings of the present study suggest that combining chitosan with EO nanoemulsion enhances its interaction with the food matrix, allowing the preservative properties of the EO to be more evenly distributed throughout the food product. This may result in increased efficacy regarding preservation and quality.

This study was limited to evaluating the effects of chitosan coating with *E. campestre* EO nanoemulsion on ostrich meat stored under refrigerated conditions for a specific duration. Factors such as cellular biochemistry and mechanisms of action at the molecular level were not assessed. Future studies should explore these aspects, along with testing different concentrations, alternative meat types, and packaging methods, to better understand the full potential and applications of this natural preservation approach.

## Author Contributions

**Faegheh RoshanNejad Moadab:** funding acquisition (equal), investigation (equal), methodology (equal), project administration (equal), resources (equal). **Maryam Azizkhani:** conceptualization (equal), data curation (equal), formal analysis (equal), project administration (equal), software (equal), supervision (equal), validation (equal), visualization (equal), writing – original draft (equal), writing – review and editing (equal).

## Ethics Statement

The authors have nothing to report.

## Conflicts of Interest

The authors declare no conflicts of interest.

## Data Availability

The data that support the findings of this study are available on request from the corresponding author.
